# Transformation Capability Optimization and Product Application Potential of *Proteatia brevitarsis* (Coleoptera: Cetoniidae) Larvae on Cotton Stalks

**DOI:** 10.3390/insects13121083

**Published:** 2022-11-24

**Authors:** Guangjie Zhang, Yeshan Xu, Shuai Zhang, Andong Xu, Zhuo Meng, Hao Ge, Jing Li, Yusheng Liu, Deying Ma

**Affiliations:** 1 Engineering Research Centre of Cotton, Ministry of Education, Key Laboratory of the Pest Monitoring and Safety Control on Crop and Forest, College of Agronomy, Xinjiang Agricultural University, 311 Nongda East Road, Urumqi 830052, China; 2 College of Plant Protection, Shandong Agricultural University, Tai’an 271018, China

**Keywords:** cotton stalks, manure, decomposition inoculant, *Proteatia brevitarsis* Lewis, biotransformation, feed, fertilizer

## Abstract

**Simple Summary:**

The Xinjiang Uyghur autonomous region is the most important area for cotton production in China, where recycling of cotton stalks (CS) as a useful resource should be encouraged. This article investigated the technical feasibility of CS as a feed and fertilizer based on the transformation of *P. brevitarsis* larvae. Decomposition inoculant, fermentation duration, and cattle manure ratio were considered the key factors affecting the transformation capability of *P. brevitarsis* larvae on CS. The research showed that 40–50% of cattle manure, 0.1% VT inoculant, and a fermentation duration of 25–30 days were the optimal technical parameters. The protein content of the larval body was as high as 52.49%, and the fat content was 11.7%. The organic matter content of frass (larvae dung-sand) was 54.8%, and the content of total nitrogen, phosphorus, and potassium (TNPK) was 9.04%, which is twice more than that of the organic fertilizer standard (NY525-2021, Beijing, China, TNPK ≥ 4.0%). The application of CS as feed (larval body) and fertilizer (larvae dung-sand) is feasible, promoting the utilization of both CS and cattle manure.

**Abstract:**

Cotton stalks (CS) are a potential agricultural biomass resource. We investigated the use of CS as a feed for *Proteatia brevitarsis* Lewis larvae and the resulting frass (larvae dung-sand) as a fertilizer. Based on a three-factor experiment (decomposition inoculant, fermentation duration, and cattle manure ratio), the optimal parameters for the transformation of CS using *P. brevitarsis* larvae were determined as 40–50% of cattle manure, the use of VT inoculant and a fermentation duration of 25–30 days. Regarding the products of the transformation, the protein content of the larval body was as high as 52.49%, and the fat content was 11.7%, which is a suitable-quality insect protein source. The organic matter content of larvae dung-sand was 54.8%, and the content of total nitrogen, phosphorus, and potassium (TNPK) was 9.04%, which is twice more than that of the organic fertilizer standard (NY525-2021, Beijing, China, TNPK ≥ 4.0%), and larvae dung-sand has the potential of fertilizer application. Therefore, CS as a feed and fertilizer based on the transformation of *P. brevitarsis* larvae is feasible, and it is a highly efficient way to promote the utilization of both CS and cattle manure.

## 1. Introduction

The Xinjiang Uyghur autonomous region is the most important area for cotton (*Gossypium hirsutum* L.) production in China. The cotton planting area is about 2.5 million hectares, and the cotton yield exceeds 5.0 million tons [1]. This area also produces cotton stalks (CS) equivalent to five times the cotton yield. Excluding the cotton leaves and root stubble, the CS yield that can be mechanically harvested is approximately 12 million tons [2]. With the characteristics of high calorific value, prominent cellulose and lignin content, and abundant nutrients, CS is used as a renewable agricultural biomass resource for energy [3,4], industrial raw materials [5], fertilizer [6], and feed [7,8]. However, more than 80% of CS is currently crushed and returned to the field directly as fertilizer [9,10]. The fertilizer effect of CS has been diminishing due to the direct return to the field in successive years. Meanwhile, the disadvantageous effects (e.g., aggravation of cotton Verticillium wilt (*Verticillium dahliae* kieb), deterioration of the soil structure) on cotton growth, yield, and quality have become more apparent [11,12,13,14,15]. For this reason, the indirect return of CS to the field has been attracting increased attention. In recent years, technologies and the utilization of micro-livestock (e.g., environmental insects, earthworms) to transform organic waste (e.g., crop residues, livestock manure) into feed and fertilizer have been attracting greater attention [16,17,18,19,20,21,22,23,24,25,26]. Micro-livestock has notable advantages in reducing greenhouse gas emissions (e.g., CO_2_, CH_4_) and promoting carbon peaking and carbon neutrality strategies [27,28,29]. In particular, the application potential of *Proteatia brevitarsis* Lewis larvae to transform crop stalks and animal manure is outstanding [30,31].

*P. brevitarsis* is an insect belonging to the genus *Protaetia*, the family Cetoniidae, and the order Coleoptera, which is widely distributed in China, Russia, North Korea, Mongolia, and other countries [32,33]. Adults are phytophagous or saprophagous, which are harmful in nature [34]. The larvae are saprophagous, which have strong transformation capability and can transform crop stalks [35,36,37], animal manure [38,39,40], edible fungus chaff [41,42,43,44] and other organic wastes efficiently. Dry larvae are a relatively high-quality protein feed ingredient with a protein content of about 50% [45,46,47,48]. Frass (larvae dung-sand) is rich in humic acids (HAs), beneficial microorganisms and nutrient elements, and it has suitable granularity and stable properties [49,50]. Dung-sand is an excellent raw material for bio-fertilizer and has shown promising effects in the cultivation of horticultural crops [51,52,53,54]. The larvae, together with the larvae of other Scarabaeoidae (i.e., *Holotrichia parallela* Motschulsky), are known as grubs. As the traditional medicine and feed insects in China and Korea, grubs have functions in anticancer [55,56], antibacterial [57], antioxidant [58], and anti-inflammation [59,60]; therefore, *P. brevitarsis* has suitable development prospects in food and feed industries [61]. On the other hand, the genome and transcriptome sequencing of *P. brevitarsis* has been completed, which lays the foundation for in-depth research and development of its resource value of *P. brevitarsis* [62,63]. In conclusion, *P. brevitarsis* has potential resources in the fields of transforming organic wastes, pharmaceutical applications, feed ingredients and organic fertilizers.

Decomposition microorganisms promote pre-decomposition and humification of materials and provide assistance to carrion feeders (e.g., earthworms, dung beetles, wood-eating beetles, the black soldier fly (*Hermetia illucens* L.), etc.) in feeding and digesting food [64,65,66,67,68,69]. Studies have shown that fermentation of lignin- and cellulose-rich organic materials with specific microbial inoculants followed by vermicomposting or insect composting can not only improve the yield of production and nutritional value of frass but also shorten the time for organic materials to become standard organic fertilizer [70,71,72,73,74,75]. Based on the previous work, this study initially screened five decomposition inoculants suitable for the pre-treatment of organic waste from the transformation of *P*. *brevitarsis* larvae [31,40]. On the other hand, the C/N ratio is essential for material decomposition [76,77,78]. This study chose cattle manure, which is plentiful in the Xinjiang region and is a better feed for *P. brevitarsis* larvae, as the auxiliary material to adjust the C/N ratio of the raw materials [79]. Previous studies have shown that fermentation duration is another key factor affecting the transformation capability of *P. brevitarsis* larvae [37,46]. We carried out a three-factor (decomposition inoculant, fermentation duration, and cattle manure ratio) five-level orthogonal experiment to explore the best technical parameters of the transformation capability for CS using *P. brevitarsis* larvae and to evaluate the application potential of the larval body as a feed ingredient and larvae dung-sand as organic fertilizer. The significance of this study is to provide a method reference for improving the transformation capability of organic waste and promoting the utilization of cotton stalks and cattle manure.

## 2. Materials and Methods

### 2.1. Experimental Site

The experimental site was located in the Industrialization Research Base of Environmental Insect Transforming Organic Waste, Xinjiang Agricultural University, in Manas County (44°13′49″ N, 86°23′3″ E), Changji Prefecture, China.

### 2.2. Experimental Materials

Cotton stalks (CS) and cattle manure were taken from farmers or herders around the base. The larvae of *P. brevitarsis* were self-reproduced in the base. Materials such as decomposition inoculants (Table 1), cucumber (*Cucumis sativus* L.) seeds (Changchun Mithorn, Xinjiang Lianchuang Seed Co., Ltd., Urumqi, China; for the determination of the seed germination index), electronic balance (LT3002, Changshu Tianliang Instrument Co., Ltd., Changshu, China) and experimental tools were purchased or previously owned.

### 2.3. Experimental Methods

#### 2.3.1. Preliminary Selection of the Optimal Combination of Decomposition Inoculant, Fermentation Duration, and Cattle Manure Ratio

CS and cattle manure were dried and crushed for use. The three-factor five-level orthogonal experiment (Table 2) of decomposition inoculant, cattle manure ratio and fermentation duration were conducted in September 2020. A total of 25 treatments were designed by IBM SPSS Statistics 23.0 (SPSS 23.0) (L25 (5^6^) orthogonal table) and recorded as A_1-5_ B_1-5_ C_1-5_. The CK groups were the CS fermented for 0, 10, 15, 20, 25, and 30 days. The initial materials for every treatment were 90 kg (dry weight, the same as below). The decomposition inoculants were added at the recommended amount. The water content (WC) of the materials was adjusted to 65 (±5)%. Then, the materials were mixed and piled into a cone shape. The ambient temperature and fermentation temperature of material pile (20 cm depth) were recorded daily. Samples were taken from 20 to 30 cm below the surface of material pile (five-point sampling method) according to the days of fermentation duration for each treatment. Each sample weighed 3 kg (fresh weight) and was frozen and stored in the refrigerator. In strict accordance with the process of turning the material pile every 5 days and sampling first and then turning the pile, and the material fermentation and sampling experiments were finished after 30 days.

The samples were thawed naturally, and each culture box (1 L) was filled with 280 g of fresh material (about 80 g dry weight), 10 larvae (the 3rd instar and 15th day) of *P. brevitarsis* were put into the box. Thereafter, the transformation experiment was carried out for 15 days. Each treatment was repeated four times. On the 16th day, weighing larvae weight gain, feed intake and dung-sand weight, the feed utilization rate, dung-sand conversion rate and mortality were calculated by Liu (2012) [80]. The optimum technical parameters were selected by making a comprehensive comparison of the transformation capability of larvae.

Calculation formula (Mass unit/mg):

Feed utilization rate = (total feed weight − remaining feed weight)/total feed weight × 100%(1)

Dung-sand conversion rate = Dung-sand weight/(feeding weight − dry larvae weight gain) × 100%(2)

Mortality = number of dead larvae/number of tested larvae × 100%(3)

#### 2.3.2. Validation of the Optimal Technical Parameters for CS as Feed and Fertilizer

The validation experiment was carried out in May 2021. The optimal combination based on the experimental results of Section 2.3.1 was A_5_B_4_C_4_: VT inoculant, the ratio of cattle manure was 40%, and the fermentation duration was 25 days. The control feed (CK) was cotton stalks fermented for 25 days, and the specific operation is referred to in Section 2.3.1. Thereafter, we determined the transformation capability data of the *P. brevitarsis* larvae to CS and verified the feasibility of the optimal technical parameters.

#### 2.3.3. Determination of Related Nutritional Indicators for CS Transformation Products as Feed and Fertilizer

The feed or fertilizer nutrition indicators of the raw materials (CS and cattle manure), fermented materials (fermented CS and A_5_B_4_C_4_ feed), and products (dry larvae and larvae dung-sand) of the optimal treatment and control were determined (refer to GB 13078-2017 and NY525-2021 standards, Beijing, China, and tested by Sichuan Weil Testing Technology Co., Ltd., Chengdu, China. The seed germination index was determined by referring to the appendix of NY525-2021, Beijing, China). To explore the application potential of CS transformation by *P. brevitarsis*.

### 2.4. Data Processing

SPSS 23.0 was used to conduct a three-factor five-level analysis of variance with repeated observations and no interaction. One-Way ANOVA was performed for the CK groups and the three factors, and Tukey’s multiple comparison analysis was performed for the differences between different treatments (*p* < 0.05). Microsoft Excel 2013 was used to record and organize data and draw tables. Sigma Plot 14 was used to draw graphs.

## 3. Results

### 3.1. Preliminary Selection of the Optimal Combination of Decomposition Inoculant, Fermentation Duration, and Cattle Manure Ratio

#### 3.1.1. Effect of Fermentation Duration on Transformation Capability to CS Using *P. brevitarsis* Larvae

As shown in Table 3, the transformation capability of the *P. brevitarsis* larvae on CS was significantly different under different fermentation duration. The optimal indexes of feed intake, larvae weight gain, and feed utilization rate were 25 days after fermentation. The dung-sand weight was the best after 20 days of fermentation, but the difference was insignificant compared with 25 days of fermentation. The dung-sand conversion rate was optimal after 15 days of fermentation, which was not significantly different from that after 20 days of fermentation. The mortality of larvae was the lowest at the 15 and 25 days of fermentation duration, and there was no significant difference among all treatments. Comprehensive analysis showed that the transformation capability of the *P. brevitarsis* larvae on CS was the best for 25 days after fermentation.

#### 3.1.2. Influence of Three Factors on the Fermentation Temperature of Materials

As shown in Table 4, under the fermentation cycle of every 5 days, the influence of the decomposition inoculant on the fermentation temperature of the material pile did not reach a significant difference level, and the overall situation was relatively stable. The influence of the ratio of cattle manure on the fermentation temperature of the material pile reached a significant difference level on the 10th, 20th, and 30th days. In the first 20 days, the fermentation temperature of the material pile at the 10% cattle manure group was the highest, and that of the 50% cattle manure group was lower. After 25 days, the temperature showed an opposite trend. In terms of fermentation duration, only 25 days of fermentation showed a significant difference level, which should be the inflection point of material fermentation temperature. After 30 days of fermentation, except for the CK group, the fermentation temperature of 25 treatments was above 30 °C, which was much higher than the ambient temperature on the same day. In the early stage, the temperature of the CK group was high, but the temperature dropped sharply after 20 days. The temperature of the 25 treatments only dropped significantly after 25 days of fermentation, which was related to the degree of material fermentation entering the later stage and also related to the low ambient temperature (the average temperature after 20 days was lower than 10 °C). The trend of temperature variation among different treatments showed that adding decomposition inoculant and cattle manure could maintain the temperature of the material pile in a high and stable range and then promote the fermentation of CS.

#### 3.1.3. Differences in the Transformation Capability of the *P. brevitarsis* Larvae on CS Considering Three Factors

Table 5 has shown that the transformation capability of the *P. brevitarsis* larvae with different decomposition inoculants was significantly different in the indexes of feed intake and weight gain but not significantly different in the other four indexes, and VT inoculant was the best. As for the factor of cattle manure ratio, 40% and 50% groups showed the best performance, and the indexes of feed intake, dung-sand weight, feed utilization rate, and dung-sand conversion rate were significantly different from the 10% and 20% groups. The transformation capability of the *P. brevitarsis* larvae was the best at 25 days and 30 days after fermentation, and the feed intake, dung-sand weight, and feed utilization rate of the third instar larvae were significantly higher than those at 10 days after fermentation. The difference in transformation capability of the larvae under the three factors provided suitable support for optimizing the technical parameters of the transformation of CS using the *P. brevitarsis*.

#### 3.1.4. Test of Inter-Subjects Effects under Three Factors

It can be seen from Table 6 that the effects of the three factors on feed intake, dung-sand weight, feed utilization rate, and dung-sand conversion rate were significantly different, while the differences in larvae weight gain and mortality were not significant. This experiment mainly analyzed four indexes with significant differences. According to the comparison of the type III sum of squares, the order of influencing factors for the feed intake was from largest to smallest: B > C > A. For the three assessment indicators of dung-sand weight, feed utilization, and dung-sand conversion rate, the order of the three effect factors was C > B > A.

#### 3.1.5. Intuitive Analysis and Tukey Test under Three Factors

**Figure 1:**
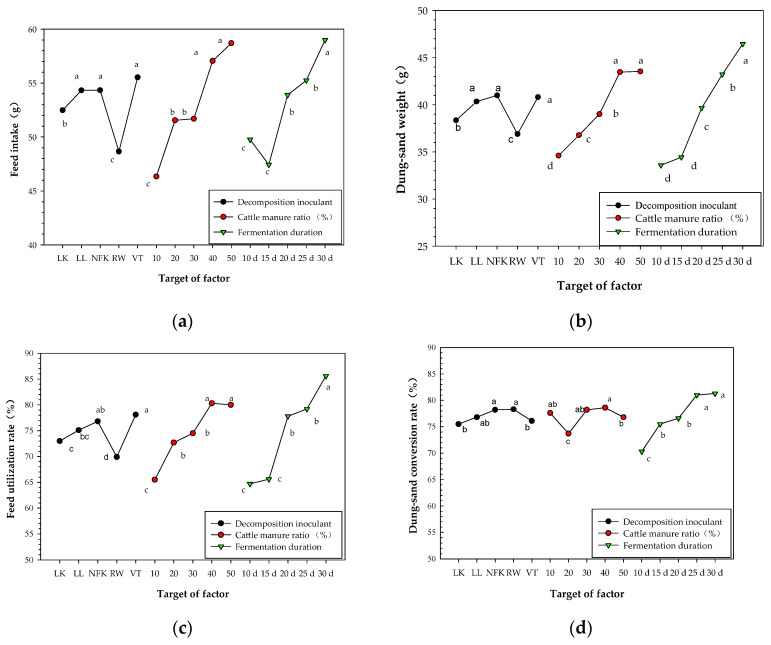
Effect of the decomposition inoculant, cattle manure ratio, and fermentation duration on the feed intake (**a**), dung-sand weight (**b**), feed utilization rate (**c**), and dung-sand conversion rate (**d**) of the 3rd instar larvae of *P. brevitarsis*. Tukey’s multiple-range tests were used for the analysis. The same factor with a different letter indicated a significant difference (*p* < 0.05, n = 20).

As can be seen from Figure 1, when the feed intake (a) and dung-sand weight (b) were used as the screening indicators, the optimal combination of the decomposition inoculant (A), cattle manure ratio (B), and fermentation duration (C) was: VT inoculant, 40% (50%) of cattle manure ratio, and 30 days of fermentation duration.

When the feed utilization rate (c) was used as the screening indicator, the optimal combination of the decomposition inoculant, cattle manure ratio, and fermentation duration was: VT inoculant, 40% (50%) of cattle manure ratio, and 30 days of fermentation duration. When the dung-sand conversion rate (d) was used as the screening indicator, the RW and NFK inoculant, 40% of cattle manure ratio, and 30 days (25 days) of fermentation duration were optimum.

According to the results of intuitive analysis and Tukey’s test (Figure 1), and referring to the results that the transformation capability of the *P. brevitarsis* larvae was the best when CS was fermented for a duration of 25 days (Table 3), the principles of minimizing cattle manure ratio, shortening fermentation duration, and reducing treatment cost were also considered. The optimal combination was A_5_B_4-5_C_4-5_ (0.1% VT inoculant, 40–50% of cattle manure ratio, and 25–30 days of fermentation duration), and A_5_B_4_C_4_ was given preference.

### 3.2. Validation of the Optimal Technical Parameters for the Transformation of CS Using P. brevitarsis Larvae

CS fermentation and transformation experiments were performed under the optimal combination (A_5_B_4_C_4_). The results are shown in Table 7.

It can be seen from Table 7 that under the optimal technology combination, the transformation capability of the *P. brevitarsis* larvae on the A_5_B_4_C_4_ feed was significantly different in feed intake, dung-sand weight, feed utilization rate, dung-sand conversion rate, and mortality with that of CK, and the feed utilization rate and dung-sand conversion rate were over 80%. Therefore, the optimal technical parameters for CS resource utilization were determined as A_5_B_4_C_4_: 0.1% VT inoculant, 40% of cattle manure ratio, and 25 days of fermentation duration. The fresh weight of fermentation material (A_5_B_4_C_4_ feed) was weighed, the water content was measured, and the yield of the material was calculated to be 62.85%. It can be concluded that 104.75 g of A_5_B_4_C_4_ feed can be obtained by adding 66.67 g of cattle manure for every 100 g of CS raw material. A total of 70.92 g of larvae dung-sand can be obtained by the third instar larvae of *P. brevitarsis*, and the weight gain of the dry larvae is 3.06 g, and 20.88 g of residue is left.

### 3.3. Determination of Relevant Nutritional Indicators of Raw Materials, Fermentation Materials, and Products

#### 3.3.1. Determination of Nutritional Indicators of Raw Materials, Fermented Materials, and Insect Bodies as Feed

It can be seen from Table 8 that the protein content of fermented CS increased by 41.9%, the crude fiber content decreased slightly, the content of gross energy (GE) was slightly increased, and the contents of crude ash and water-soluble chlorides increased greatly. The crude protein (CP) content of A_5_B_4_C_4_ feed reached 13.18%, which was slightly lower than 14.16% of cow manure and was 1.29 and 1.84 times that of the fermented and unfermented CS. Compared to the fermented CS, the A_5_B_4_C_4_ feed significantly reduced crude fiber content, increased the crude ash and water-soluble chloride content, and decreased GE. The content of free gossypol (FG) in fermented materials was about 50% lower than that in raw materials. The FG in the A_5_B_4_C_4_ feed was not detected in the larvae of *P. brevitarsis* (detection limit is 20 mg/kg). The protein (52.49%) and fat (11.7%) content of the *P. brevitarsis* dry larvae were much higher than those of the A_5_B_4_C_4_ feed, while the content of crude fiber was only 6.1%, and the content of water-soluble chloride was lower than that of the A_5_B_4_C_4_ feed. The GE (19.20 KJ/g) was intermediate between carbohydrate (17.5 KJ/g) and protein (23.64 KJ/g). The insect-microorganism composite systems can improve the nutrition indicators of CS as a feed, and the larval body was 7.31, 19.50, and 1.16 times higher than that of CS in protein, fat, and total energy and more than 50% lower in FG, and the content of crude fiber is only 1/6 of CS.

#### 3.3.2. Determination of Nutritional Indicators for Raw Materials, Fermentation Materials, and Larvae Dung-Sand as Organic Fertilizer

As shown in Table 9, the organic matter (OM) content of the six materials was above 54%, and the CS was the highest (67%). Their total nutrient (TNPK) content was more than 4.0%. The total nutrient (TNPK) and potassium (TK) content of the A_5_B_4_C_4_ feed were 9.04% and 4.44%. For the germination index (GI), the unfermented CS (47.09%) and manure (66.87%) had certain toxicity to seed germination, the GI of the remaining four materials was more than 70%, indicating that it was non-toxic to seed germination, and the GI of fermented CS was 102.88, which could promote the seed germination. The pH value of the six materials ranged from 6.6 to 9.5, and it was neutral to alkaline overall. OM decreased, HAs and GI increased first and then decreased, and TNPK, water-soluble chloride, and pH values increased in the insect-microorganism composite process from raw materials to fermentation materials and then to larvae dung-sand. In addition to pH value, two kinds of fermentation materials and two kinds of larvae dung-sand were in line with the latest standards of organic fertilizers in China in terms of OM, NPK, and GI (NY525-2021, NPK ≥ 4%, DOM ≥ 30%, GI ≥ 70%, pH 5.5–8.5).

## 4. Discussion

This study showed that for every 100 g of cotton stalks supplemented with 66.67 g of manure, 104.75 g of A_5_B_4_C_4_ feed was obtained, and 70.92 g of dung-sand was obtained after transformation by the third instar larvae of *P. brevitarsis*. The weight gain of the dry larvae was 3.06 g, and 20.88 g of residue remained. The larvae of the *P. brevitarsis* had a 27.41-fold ability to transform fermented materials (FCR = weight of feed intake/weight gained), which was nearly six times higher than that of the black soldier fly (FCR = 4.5), and had a higher feed utilization rate (80.07% ± 0.65%) and dung-sand conversion rate (84.55% ± 0.53%) [73]. Compared with other dung beetles, *P. brevitarsis* are more suitable to perform the ecological function of converting organic waste in concentrated agricultural and livestock areas because of their high reproductive ability and their tendency to gather to lay eggs and feed [34,46,65,81,82]. A previous study showed that the ratio of material surface/volume was positively correlated with the fermentation effect, and future work could improve the transformation capability of *P. brevitarsis* larvae on cotton stalks by reducing the crushing particle size and other measures [75,83]. Previous studies have only focused on the transformation efficiency of the larvae of *P. brevitarsis* for fermented material; this study also paid specific attention to the productivity from raw materials to fermented materials. According to the calculation results, the productivity of the A_5_B_4_C_4_ feed was 62.85%, which was theoretically higher than the rate of traditional organic fertilizer production methods, as judged by the 25 days required for fermentation duration [70,71,72,84,85]. The productivity of fermentation materials can provide data support for the productivity from raw materials to dry larvae and dung-sand.

Some researchers have shown that long-term feeding of excessive amounts of non-detoxified cotton by-products (e.g., cotton leaves, cottonseed meal, and cotton stalks) to vertebrates can lead to the accumulation of free gossypol (FG) in the fed animals, causing poisoning and acute respiratory distress, anorexia, fatigue, and even death [86,87,88]. This has hindered the application of cotton stalks as fodder. In this study, the contents of FG in cotton stalks, cattle manure, fermented cotton stalks, and A_5_B_4_C_4_ feed were 96, 114, 47, and 59 mg/kg. The decomposition of inoculant fermentation can significantly reduce the content of FG, which is consistent with the reduction of FG content in feed through fermentation in previous studies [89,90,91]. Interestingly, no FG was detected in the *P. brevitarsis* larvae after feeding on the A_5_B_4_C_4_ feed, indicating that the FG did not accumulate in the larvae, which may be related to the larvae-degrading FG through feeding and metabolism or the short feeding time. The specific reason is the direction of future research. The insect-microorganism composite systems can undoubtedly reduce the content of FG, and the study of its degradation mechanism may provide a reference for reducing the toxicity of FG in livestock feeding on cotton by-products. The protein and fat content of the larval body were 52.49% and 11.7%. It was a suitable-quality, high-protein, insect-derived feed ingredient [92,93], and the nutrient composition of the larvae of *P. brevitarsis* was consistent with previous studies [46,48,94]. In conclusion, it is feasible to transform cotton stalks to dry larvae feed.

Organic matter (OM) and total nutrients (TNPK) are the most commonly used indicators for evaluating organic fertilizer. This study showed that the OM and TNPK indicators of cotton stalks and manure met the Chinese organic fertilizer standards (NY525-2021, China), but they cannot be applied directly as organic fertilizers [95,96]. Therefore, the evaluation of whether the materials can be used as organic fertilizers should refer to other indicators, such as the germination index (GI), humic acids (HAs), the number of beneficial microorganisms, and so on [44,49,50,69]. Furthermore, the application effect on crops is the core criterion for evaluating the quality of an organic fertilizer [97,98,99]. The larvae dung-sand obtained in this study was much better than the Chinese organic fertilizer standard in terms of OM, TNPK, and other nutrition indicators. However, the high pH value and water-soluble chloride content may be the reason for the low GI of seeds. The quality of larvae dung-sand as organic fertilizer can be improved by adjusting pH and other measures. On the other hand, larvae dung-sand has the characteristics of regular particles and uniform texture, which is easy to process and use and can be processed into prototype flower fertilizer [31]. In cash crops, it can be applied by sowing while fertilizing or using leaching solution drip irrigation, which has the potential to be used as dung-sand-based organic fertilizer [44,54,69].

## 5. Conclusions

The optimum technical parameters for transforming cotton stalks using *P. brevitarsis* larvae were supplementation with 40–50% of cattle manure, the addition of 0.1% VT inoculant, and a fermentation duration of 25–30 days. The dry larvae are a high-protein feed ingredient from an insect-derived, which can be fed and recycled into the ecological breeding industry. The larvae dung-sand is rich in nutrition and has the potential for fertilizer application. This study preliminarily proves the feasibility of cotton stalk feeding and fertilizer dual-use technology based on the transformation of *P. brevitarsis* larvae. It possesses substantial significance for both theoretical and practical investigations related to boosting the recycling utilization of cotton stalks and cattle manure.

## Figures and Tables

**Table 1:** Introduction and instructions for decomposition inoculants.

**Decomposition Inoculants**	**Brand and Production Company**	**Main Functional Bacteria**	**Effective Number of Viable Bacteria (100 million/g)**	**Recommended Dosage (kg/t)**
LK	Organic material decomposing inoculant, stalks type, Zhongnong Lvkang Biotechnology Co., Ltd., Beijing, China	*Bacillus*, *Trichoderma,* and yeast	8	0.5
LL	Organic fertilizer decomposing inoculant, Shandong Lvlong Biotechnology Co., Ltd., Zhucheng, China	*Bacillus subtilis*, *Bacillus licheniformis*, yeast, and *Trichoderma viride*	200	10
NFK *	Organic material decomposing inoculant, Henan NongFukang Biotechnology Co., Ltd., Zhengzhou, China	Mainly *Bacillus licheniformis*, *Candida utilis*, *Bacillus subtilis*, *Lactobacillus,* and *Enterococcus-like bacteria*	0.1	30
RW	RW decomposing inoculant, stalks type, Hebi Renyuan Biological Co., Ltd., Hebi, China	Bacteria (*Bacillus subtilis, Bacillus licheniformis, and Bacillus jelly*), filamentous fungi, and yeast	100	10
VT	VT-1000, stalks type, Beijing VOTO Biotechnology Co., Ltd., Beijing, China	*Bacillus*, actinomycetes, lactic acid bacteria, and molds	200	1

* Decomposition inoculants need to be activated in advance.

**Table 2:** Orthogonal experimental factors and levels.

Level	Factor		
	Decomposing Inoculants (A)	Cattle Manure Ratio (B/%)	Fermentation Duration (C/d)
1	LK	10	10
2	LL	20	15
3	NFK	30	20
4	RW	40	25
5	VT	50	30

**Table 3:** Transformation capability of the 3rd instar larvae of *P. brevitarsis* on CS under different fermentation durations.

Fermentation Duration (d)	Feed Intake (g)	Larvae Weight Gain (g)	Dung-Sand Weight (g)	Feed Utilization Rate (%)	Dung-Sand Conversion Rate (%)	Mortality (%)
0	48.50 ± 1.18a	1.89 ± 0.09a	19.16 ± 0.28d	54.78 ± 1.33b	41.17 ± 1.27d	5.00 ± 2.89a
10	37.68 ± 1.13c	1.81 ± 0.10a	28.32 ± 0.30c	44.11 ± 1.32c	79.17 ± 2.65ab	2.50 ± 2.50a
15	36.33 ± 0.44c	1.82 ± 0.10a	30.91 ± 0.31b	45.14 ± 0.55c	89.57 ± 0.63a	0.00 ± 0.00a
20	49.11 ± 0.64a	2.04 ± 0.13a	36.98 ± 0.60a	62.83 ± 0.81a	78.54 ± 0.48ab	2.50 ± 2.50a
25	49.24 ± 0.46a	2.18 ± 0.10a	35.24 ± 0.61a	64.66 ± 0.60a	74.86 ± 0.85c	0.00 ± 0.00a
30	41.58 ± 0.50b	1.92 ± 0.04a	32.30 ± 0.75b	55.03 ± 0.67b	81.45 ± 1.41b	2.50 ± 2.50a

Data in the table are mean ± standard error (SE). Different letters in the same column indicate a significant difference (*p* < 0.05). The same is below.

**Table 4:** Effect of the decomposition inoculant, cattle manure ratio, and fermentation duration on the fermentation temperature of the material pile.

Factor and Level	Temperature (°C)						
	1 d	5 d	10 d	15 d	20 d	25 d	30 d
Decomposing inoculants (A)							
LK	41.80 ± 2.51a	55.58 ± 3.78a	48.50 ± 1.74a	48.88 ± 1.31a	47.72 ± 0.52a	47.88 ± 1.35a	43.30 ± 2.04a
LL	41.04 ± 2.07a	53.18 ± 2.75a	48.20 ± 1.69a	46.08 ± 1.64a	47.86 ± 1.39a	49.40 ± 1.79a	43.60 ± 0.46a
NFK	42.98 ± 2.74a	52.68 ± 3.94a	48.16 ± 1.92a	48.94 ± 2.44a	48.94 ± 1.85a	49.24 ± 1.81a	44.66 ± 1.32a
RW	41.06 ± 1.89a	50.24 ± 3.63a	48.96 ± 2.32a	46.46 ± 0.92a	46.64 ± 2.11a	47.26 ± 1.75a	42.58 ± 1.40a
VT	40.76 ± 1.22a	54.82 ± 1.35a	49.78 ± 2.43a	48.78 ± 1.03a	46.86 ± 1.34a	48.52 ± 1.67a	42.14 ± 2.86a
Cattle manure ratio (B/%)							
10	43.80 ± 2.13a	58.34 ± 1.74a	53.22 ± 1.70a	50.36 ± 1.06a	51.10 ± 1.35a	50.30 ± 1.96a	38.78 ± 2.00b
20	40.86 ± 2.98a	55.64 ± 2.89a	49.50 ± 1.18ab	47.76 ± 1.24a	48.20 ± 0.22ab	50.96 ± 1.48a	45.20 ± 0.98a
30	42.88 ± 1.18a	50.70 ± 2.77a	46.42 ± 1.71ab	47.66 ± 1.13a	47.24 ± 1.39ab	46.76 ± 1.70a	43.48 ± 1.71ab
40	41.42 ± 1.80a	53.98 ± 3.87a	48.36 ± 1.95ab	44.92 ± 2.30a	45.08 ± 1.41b	46.20 ± 1.07a	42.98 ± 1.05ab
50	38.68 ± 1.43a	47.84 ± 2.38a	46.10 ± 1.43b	48.44 ± 1.14a	46.40 ± 1.35ab	48.08 ± 0.68a	45.84 ± 0.78a
Fermentation duration (C/d)							
10	39.68 ± 1.69a	51.14 ± 2.01a	49.40 ± 2.21a	46.30 ± 1.53a	47.78 ± 0.35a	48.22 ± 1.61ab	43.74 ± 0.97a
15	42.36 ± 2.23a	52.26 ± 2.65a	47.00 ± 1.60a	47.98 ± 1.00a	47.62 ± 0.70a	45.14 ± 0.42b	40.72 ± 1.47a
20	41.74 ± 2.06a	59.20 ± 1.40a	48.92 ± 2.27a	48.10 ± 2.36a	48.64 ± 2.51a	48.28 ± 2.01ab	43.60 ± 1.77a
25	40.48 ± 2.14a	50.60 ± 3.50a	48.88 ± 2.25a	47.70 ± 1.61a	46.88 ± 1.79a	49.12 ± 0.75ab	42.60 ± 2.58a
30	43.38 ± 2.27a	53.30 ± 4.41a	49.40 ± 1.63a	49.06 ± 1.35a	47.10 ± 1.46a	51.54 ± 1.53a	45.62 ± 1.08a
CK	48.50	57.60	52.90	45.60	40.10	21.90	17.30
Ambient temperature	16.50	15.50	20.50	18.50	12.00	9.50	6.50

**Table 5:** Effect of the decomposition inoculant, cattle manure ratio, and fermentation duration on the transformation capability of the 3rd instar larvae of *P. brevitarsis*.

Factor and Level	Feed Intake (g)	Larvae Weight Gain (g)	Dung-Sand Weight (g)	Feed Utilization Rate (%)	Dung-Sand Conversion Rate (%)	Mortality (%)
Decomposing inoculants (A)						
LK	52.48 ± 2.16ab	1.833 ± 0.043ab	38.35 ± 1.95a	72.99 ± 3.02a	75.46 ± 1.37a	0.50 ± 0.50a
LL	54.32 ± 1.33ab	1.928 ± 0.051ab	40.34 ± 1.56a	75.14 ± 2.57a	76.78 ± 1.89a	1.00 ± 0.69a
NFK	54.33 ± 1.22ab	1.886 ± 0.048ab	40.99 ± 0.99a	76.81 ± 1.78a	78.19 ± 0.81a	1.00 ± 0.69a
RW	48.66 ± 1.69b	1.753 ± 0.054b	36.88 ± 1.57a	69.85 ± 3.08a	78.32 ± 1.07a	1.50 ± 1.09a
VT	55.53 ± 1.18a	1.949 ± 0.051a	40.81 ± 1.20a	78.10 ± 1.81a	76.06 ± 1.37a	1.50 ± 0.82a
Cattle manure ratio (B/%)						
10	49.76 ± 1.50bc	1.799 ± 0.060a	33.58 ± 1.03c	64.66 ± 2.39c	70.33 ± 1.37c	2.50 ± 1.23a
20	47.43 ± 1.96c	1.846 ± 0.058a	34.43 ± 1.49c	65.60 ± 2.43c	75.53 ± 0.87b	1.00 ± 0.69a
30	53.89 ± 1.52ab	1.895 ± 0.042a	39.68 ± 0.95b	77.80 ± 1.86b	76.64 ± 1.15b	1.00 ± 0.69a
40	55.25 ± 0.61a	1.905 ± 0.047a	43.22 ± 0.79ab	79.19 ± 0.63ab	80.97 ± 0.90a	0.50 ± 0.50a
50	58.99 ± 0.71a	1.904 ± 0.046a	46.45 ± 0.71a	85.64 ± 0.94a	81.35 ± 0.63a	0.50 ± 0.50a
Fermentation duration (C/d)						
10	46.34 ± 2.15c	1.863 ± 0.068a	34.60 ± 1.71b	65.45 ± 3.73b	77.62 ± 0.62a	1.00 ± 1.00a
15	51.54 ± 1.15b	1.906 ± 0.040a	36.77 ± 1.50b	72.68 ± 2.13ab	73.68 ± 1.68a	1.00 ± 0.69a
20	51.69 ± 1.20b	1.821 ± 0.040a	39.00 ± 1.23ab	74.47 ± 2.18a	78.16 ± 1.48a	2.00 ± 0.92a
25	57.05 ± 0.81a	1.843 ± 0.047a	43.46 ± 0.98a	80.28 ± 1.28a	78.58 ± 0.74a	0.50 ± 0.50a
30	58.70 ± 0.75a	1.917 ± 0.056a	43.53 ± 0.91a	80.01 ± 1.12a	76.78 ± 1.63a	1.00 ± 0.69a

**Table 6:** Tests of inter-subjects effects.

Source	Dependent Variable	Type III Sum of Squares	df	Mean Square	F	Sig.
Corrected Model	Feed intake	4186.996 ^a^	12	348.916	29.758	0.000
	Larval weight gain	0.806 ^b^	12	0.067	1.353	0.204
	Dung-sand weight	3987.502 ^c^	12	332.292	57.961	0.000
	Feed utilization rate	1.049 ^d^	12	0.087	31.856	0.000
	Dung-sand conversion rate	0.206 ^e^	12	0.017	9.815	0.000
	Mortality	0.009 ^f^	12	0.001	0.614	0.825
Decomposition inoculant (A)	Feed intake	581.020	4	145.255	12.388	0.000
	Dung-sand weight	256.548	4	64.137	11.187	0.000
	Feed utilization rate	0.085	4	0.021	7.760	0.000
	Dung-sand conversion rate	0.013	4	0.003	1.848	0.127
Cattle manure ratio (B)	Feed intake	1940.292	4	485.073	41.371	0.000
	Dung-sand weight	1272.551	4	318.138	55.492	0.000
	Feed utilization rate	0.298	4	0.074	27.140	0.000
	Dung-sand conversion rate	0.031	4	0.008	4.368	0.003
Fermentation duration(C)	Feed intake	1665.684	4	416.421	35.516	0.000
	Dung-sand weight	2458.403	4	614.601	107.204	0.000
	Feed utilization rate	0.666	4	0.166	60.666	0.000
	Dung-sand conversion rate	0.163	4	0.041	23.230	0.000
Error	Feed intake	1020.076	87	11.725		
	Larval dry weight	4.318	87	0.050		
	Dung-sand weight	498.772	87	5.733		
	Feed utilization rate	0.239	87	0.003		
	Dung-sand conversion rate	0.153	87	0.002		
	Mortality	0.109	87	0.001		
Corrected total	Feed intake	5207.072	99			
	Larval dry weight	5.124	99			
	Dung-sand weight	4486.274	99			
	Feed utilization rate	1.288	99			
	Dung-sand conversion rate	0.359	99			
	Mortality	0.118	99			

^a^. R squared = 0.804 (adjusted R squared = 0.777); ^b^. R squared = 0.157 (adjusted R squared = 0.041). ^c^. R squared = 0.889 (adjusted R squared = 0.873); ^d^. R squared = 0.815 (adjusted R squared = 0.789). ^e^. R squared = 0.575 (adjusted R squared = 0.517); ^f^. R squared = 0.078 (adjusted R squared = −0.049).

**Table 7:** Transformation capability of the 3rd instar larvae of *P. brevitarsis* under the optimal combination.

Treatments	Feed Intake (g)	Larvae Weight Gain (g)	Dung-Sand Weight (g)	Feed Utilization Rate (%)	Dung-Sand Conversion Rate (%)	Mortality (%)
CK	51.92 ± 0.37	2.030 ± 0.102	40.48 ± 0.39	64.90 ± 0.46	81.13 ± 0.38	2.50 ± 2.50 *
A_5_B_4_C_4_	64.06 ± 0.52 *	2.338 ± 0.049	52.19 ± 0.60 *	80.07 ± 0.65 *	84.55 ± 0.53 *	0.00 ± 0.00

Using independent sample *T*-test, * means significantly different (Tukey test, *p* < 0.05, n = 4).

**Table 8:** Key nutritional indicators for raw materials, fermented materials, and dry larvae as feed ingredients.

Material Types	WC (%)	CP (%)	Crude Fat (%)	Crude Fiber (%)	Crude Ash (%)	Water-Soluble Chloride (%)	FG (mg/kg)	GE (KJ/g)
CS	8.6	7.18	0.6	43.3	5.1	0.40	96	16.57
Cattle manure	79.2	14.16	0.6	27.4	17.6	1.20	114	14.74
Fermented CS	69.7	10.19	0.3	43.2	9.6	0.75	47	17.1
A_5_B_4_C_4_ feed	71.2	13.18	0.3	34.7	15.9	1.60	59	15.32
Dry larvae	72.0	52.49	11.7	6.1	15.6	1.00	-	19.2

**Table 9:** Main nutritional indicators for raw materials, fermentation materials, and larvae dung-sand as organic fertilizer.

Material Types	WC (%)	OM (%)	HAs (%)	TN (%)	TP (%)	TK (%)	TNPK (%)	pH	Water- Soluble Chloride (%)	GI (%)
CS	8.6	67.0	1.06	1.29	0.99	2.35	4.63	6.6	0.40	47.09
Manure	79.2	58.9	1.59	2.3	1.29	2.18	5.77	8.9	1.20	66.87
Fermented CS	69.7	65.9	2.31	2.23	0.42	3.84	6.49	9.3	0.75	102.88
A_5_B_4_C_4_ feed	71.2	59.5	1.82	2.54	1.16	4.13	7.83	9.5	1.60	98.73
CS-based larvae dung-sand	65.6	61.3	1.38	2.68	0.87	4.55	8.1	9.4	0.95	77.35
A_5_B_4_C_4_d feed-based larvae dung-sand	68.7	54.8	0.81	2.93	1.67	4.44	9.04	9.2	1.60	75.90

## Data Availability

Raw data used in this study are available on request from the authors.

## References

[1] China National Bureau of Statistics (2020). Announcement of the National Bureau of Statistics on Cotton Production in 2020. http://www.stats.gov.cn/tjsj/zxfb/202012/t20201218_1810113.html.

[2] Yuyun B. (2010). Study on Straw Resources Evaluation and Utilization in China. Ph.D. Thesis.

[3] Yingquan C., Haiping Y., Xianhua W., Shihong Z., Hanping C. (2012). Biomass-based pyrolytic polygeneration system on cotton stalk pyrolysis: Influence of temperature. Bioresour. Technol..

[4] Zhipu W., Like X., Kai L., Jian W., Henan Z., Qiang S., Xinqian S. (2019). Co-pyrolysis of sewage sludge and cotton stalks. Waste Manag..

[5] Qingyue W., Nuerjiamali T. (2020). Polyurethane foams and bio-polyols from liquefied cotton stalk agricultural waste. Sustainability.

[6] Qi W., Sheng X., Moyong Z., Maonan Y., Huijun L., Zhixing L. (2017). Operating procedures for high-yield cultivation of *Ganoderma lucidum* using cotton stalks. Cotton Sci..

[7] Guoqing Z., Qiujiang L., Changjiang Z., Fengming L., Jirong Z. (2018). Study on the nutritional value of cotton stalks and their effects on the digestion and metabolism of nutrients, growth and mutton safety of sheep. J. Anim. Nutr..

[8] Xiaofang Z., Rui G., Junyu Z. (2020). Research progress on feed utilization of cotton straws in ruminants. China Grass-Feed. Livest..

[9] Pengpeng Z., Shou-zhen X., Guojuan Z., Xiaozhen P., Jin W., Wangfeng Z. (2019). Carbon cycle in response to residue management and fertilizer application in a cotton field in arid Northwest China. J. Integr. Agric..

[10] Jing W., Bing C., Jiliang W., Yongtao L., Min W., Yong S., Huanyong H., Fangyong W. (2021). Effects of different mechanized methods of straw returning to the field on growth, yield and quality of cotton. Agric. Res. Arid Areas.

[11] Wright A.L., Hons F.M., Lemon R.G., Mark L., McFarland M.L., Nichols R.L. (2007). Stratification of nutrients in soil for different tillage regimes and cotton rotations. Soil Tillage Res..

[12] ChunL H. (2010). Temporal and Spatial Variation of Soil Nutrients of Long-Term Monocultural Cotton Field and Sustainable Utilization in Xinjiang. Ph.D. Thesis.

[13] Tesio F., Vidotto F., Ferrero A. (2012). Allelopathic persistence of *Helianthus tuberosus* L. residues in the soil. Sci. Hortic..

[14] Endeshaw S.T., Lodolini E.M., Neri D. (2015). Effects of olive shoot residues on shoot and root growth of potted olive plant lets. Sci. Hortic..

[15] Yanbin L., Qin Z. (2016). Effects of naturally and microbially decomposed cotton stalks on cotton seedling growth. Arch. Agron. Soil Sci..

[16] Subramanian S., Sivarajan M., Saravanapriya S. (2010). Chemical changes during vermicomposting of sago industry solid wastes. J. Hazard. Mater..

[17] Arnold V.H., Joost V.I., Harmke K., Esther M., Afton H., Giulia M., Paul V. (2013). Edible Insects: Future Prospects for Food and Feed Security.

[18] Cickova H., Newton G.L., Lacy R.C., Kozanek M. (2015). The use of fly larvae for organic waste treatment. Waste Manag..

[19] YuSheng L. (2015). Scientific basis and technology system of macro agriculture circle economy. Renew. Resour. Circ. Econ..

[20] Lim S.L., Lee L.H., Wu T.Y. (2016). Sustainability of using composting and vermicomposting technologies for organic solid waste biotransformation: Recent overview, greenhouse gases emissions and economic analysis. J. Clean. Prod..

[21] Kilic E. (2018). Environmental friendly insects is *Tenebrio molitor* (Coleoptera Tenebrionidae). Adv. Ecol. Res..

[22] Naseer H., Shahid A. (2018). Efficacy of the vermicomposts of different organic wastes as “Clean” fertilizers: State-of-the-art. Sustainability.

[23] Soobhany N. (2019). Insight into the recovery of nutrients from organic solid waste through biochemical conversion processes for fertilizer production: A review. J. Clean. Prod..

[24] Kawasaki K., Kawasaki T., Hirayasu H., Matsumoto Y., Fujitani Y. (2020). Evaluation of fertilizer value of residues obtained after processing household organic waste with black soldier fly larvae (*Hermetia illucens*). Sustainability.

[25] Rumbos C.I., Mente E., Karapanagiotidis I.T., Vlontzos G., Athanassiou C.G. (2021). Insect-based feed ingredients for aquaculture: A case study for their acceptance in Greece. Insects.

[26] Xinyue W., Yanhong C., Shaolang H., Kun Z., Shangshu H., Binqiang W., Qianru H. (2022). Study on earthworm transformation technology based on the fertilizer utilization of kudzu slag. J. Agric. Resour. Environ..

[27] Arnold V.H., Dennis G.A., Oonincx B. (2017). The environmental sustainability of insects as food and feed. A review. Agron. Sustain. Dev..

[28] Mertenat A., Diener S., Zurbrugg C. (2019). Black soldier fly biowaste treatment-assessment of global warming potential. Waste Manag..

[29] Borkent S., Hodge S. (2021). Glasshouse evaluation of the black soldier fly waste product HexaFrass™ as an organic fertilizer. Insects.

[30] Xiaoyan T., Fuping S., Jie Z., Rongmei L., Xingpeng Z., Jiangyan D., Changlong S. (2017). Diversity of gut bacteria in larval *Protaetia brevitarsis* (Coleoptera: Scarabaedia) fed on corn stalk. Acta Entomol. Sin..

[31] Guangjie Z. (2019). Studies on the Transformation Techniques of Organic Waste Using *Protaetia brevitarsis* (Coleoptera: Cetoniidae). Master’s Thesis.

[32] Wenzhen M. (1995). Chinese Economic Entomology (Coleoptera, Cetoniidea).

[33] Baozhong J., Shuwen L., Kai Z. (2011). Entomological Basis and Common Species Identification.

[34] Tao L., Deying M., Song Q., Yong W. (2010). A study on hosts and the occurrence regularity of *Protaetia brevitarsis* Lewis in west suburb of Urumqi. Xinjiang Agric. Sci..

[35] Cheng Y., Yusheng L., Xiaoyan X., Li Z. (2015). The study on the effect of *Protaetia brevitaris* Lewis larvae transformation the corn straw. J. Environ. Entomol..

[36] Yusheng L., Dapeng Z. (2015). Study on the model of microcirculation farm and ranch on the corn straw transformed by larval of *Protaetia brevitarsis* Lewis. J. Anhui Agric. Sci..

[37] Guangjie Z., Qian W., Yusheng L., Zeng’an L. (2019). Study on the transformation capability of four materials in different fermentation cycles fed by *Protaetia brevitarsis* (Coleoptera: Cetoniidae) larvae. J. Shandong Agric. Univ..

[38] Liu Y., Guangjie Z., Tao X., Lianjun Z., Song Q., Deying M., Yusheng L. (2019). Study on the conversion capacity different agricultural organic wastes by the larvae of the *Protaetia brevitarsis* Lewis. Xinjiang Agric. Sci..

[39] Yong G. (2020). Investigation on Agricultural Organic Waste Resources and Exploration on Conversion Mode of Agricultural Organic Waste Resources by Environment-Friendly Insects in Yuncheng County. Master’s Thesis.

[40] Tao X., Guangjie Z., Liu Y., Song Q., Deying M., Yusheng L. (2021). Technology for breeding *Protaetia brevitarsis* Lewis indoors and outdoors. Chin. J. Appl. Entomol..

[41] Qian Z. (2015). Study on the Biology of *Protaetia brevitarsis* (Lewis) Feeding on Oyster Mushroom Bran. Master’s Thesis.

[42] Chenke S. (2018). Study on the Recycling Mode of “Wheat Straw-*Stropharia rugosoannulata*-*Protaetia brevitarsis*”. Master’s Thesis.

[43] Seul-Bi L., Jong-Won K., Sung-Mun B., Yeon-Hyeon H., Heung-Su L., Byeong-Jeong L., Kwang-Pyo H., Chung-Gyoo P. (2018). Evaluation of spent mushroom substrates as food for white spotted flower chafer, *Protaetia brevitarsis* seulensis (Coleoptera: Cetoniidae). Korean J. Appl. Entomol..

[44] Panpan W., Yimei L., Deqiang L., Lili G., Chunqin L., Jie Z., Changlong S., Rongmei L. (2020). *Protaetia brevitarsis* larvae can feed on and convert spent mushroom substrate from *Auricularia auricula* and *Lentinula edodes* cultivation. Waste Manag..

[45] Cheng Y., Yusheng L., Xiaoyan X., Jianwei Z. (2014). Analysis and evaluation of resource components of *Protaetia brevitarsis* (Lewis) larvae. J. Shandong Agric. Univ..

[46] Guangjie Z., Qian W., Yusheng L. (2020). Biology under artificial condition and utilization potential of *Protaetia brevitarsis* (Coleoptera: Cetoniidae). J. Environ. Entomol..

[47] Seonmin L., Yun-Sang C., Kyung J., Tae-Kyung K., Hae-In Y., Samooel J. (2020). Quality characteristics and protein digestibility of *Protaetia brevitarsis* larvae. J. Anim. Sci..

[48] Youn-Kyung H., Sam-Woong K., Dong-Heon S., Hyun-Wook K., Il-Suk K. (2021). Nutritional Composition of White-Spotted Flower Chafer (*Protaetia brevitarsis*) Larvae Produced from Commercial Insect Farms in Korea. Food Sci. Anim. Resour..

[49] Yimei L., Tong F., Lili G., Yu S., Haiyan C., Fushun L., Chunqin L., Fuping S., Jie Z., Changlong S. (2019). *Protaetia brevitarsis* larvae can efficiently convert herbaceous and ligneous plant residues to humic acids. Waste Manag..

[50] Huina X., Peiwen G., Baohai D., Lili G., Kui W., Kun H., Jie Z., Tianpei H., Changlong S. (2022). Characterization of microorganisms from *Protaetia brevitarsis* larva frass. Microorganisms.

[51] Fushun L., Xiaojie F., Guocheng X., Yu W., Qinglei W. (2018). The effects of *Protaetia brevitarsis* larva manure application on the growth of cherry radish. Hubei Agric. Sci..

[52] Deqiang L., Qinglei W., Yu W., Changlong S., Yue Z., Chunqin L. (2019). Effect of *Protaetia brevitarsis* Lewis larvae dung on development of pepper seedling stage under low temperature. North. Hortic..

[53] Xiang W., Congyong H., Ruijie C., Xiaoyan X., Jinlong W., Xiaobo W. (2019). Influence of frass organic manure on tomato growth and quality. North. Hortic..

[54] Kyong-Hee J., Jong-Won K., Seul-Bi L., Da-Hyun J., Byung-Man Y., Sung-Mun B., Young-Ho C., Young Han L., Dong-Cheol S. (2022). Effects of *Protaetia brevitarsis* larvae manure application on lettuce growth and soil chemical properties. Korean J. Soil. Sci. Fert..

[55] Hua J., Shu S., Baiyan Y., Wanshan Y., Tiefeng J. (2008). Effects of the grub extract on apoptosis of MCF-7 human breast cancer cell line. Chin. J. Pathophysiol..

[56] Ahn E.M., Myung N.Y., Jung H.A., Kim S.J. (2019). The ameliorative effect of *Protaetia brevitarsis* larvae in HFD-induced obese mice. Food Sci. Biotechnol..

[57] Mingxu X., Guofu G., Shouyun Y., Jie S., Chongxing Z., Chunhua X., Yi L., Keyun Z. (2008). Isolation and purification of antibacterial materials from *Protaetia brevitarsis* (Coleoptera) Larva. Life Sci. Res..

[58] Hwa-Jin S., Chul K. (2012). Antioxidant activity of aqueous methanol extracts of *Protaetia brevitarsis* Lewis (Coleoptera: Scarabaedia) at different growth stages. Nat. Prod. Res..

[59] Eunjung L., Jin-Kyoung K., Soyoung S., Ki-Woong J., Juneyoung L., Dong-Gun L., Jae-Sam H., Yangmee K. (2011). Enantiomeric 9-mer peptide analogs of protaetiamycine with bacterial cell selectivities and anti-inflammatory activities. J. Pept. Sci..

[60] Minglu Q. (2020). Study on Extraction, Separation, Purification and Anti-Inflammatory Property of *Protaetia brevitarsis* Lewis Larvae Protein. Master’s Thesis.

[61] Nikkhah A., Van Haute S., Jovanovic V., Jung H., Dewulf J., Cirkovic Velickovic T., Ghnimi S. (2021). Life cycle assessment of edible insects (*Protaetia brevitarsis* Seulensis larvae) as a future protein and fat source. Sci. Rep..

[62] Zhongjie L., Miaomiao M., Shasha L., Deng B. (2019). The transcriptome analysis of *Protaetia brevitarsis* Lewis larvae. PLoS ONE.

[63] Kui W., Pengpeng L., Yongyang G., Chunqin L., Qinglei W., Jiao Y., Jie Z., Lili G., Changlong S. (2019). De novo genome assembly of the white-spotted flower chafer (*Protaetia brevitarsis*). GigaScience.

[64] Xiangzhen L., Brune A. (2005). Digestion of microbial biomass, structural polysaccharides, and protein by the humivorous larva of *Pachnoda ephippiata* (Coleoptera: Scarabaeidae). Soil Biol. Biochem..

[65] Manning P., Slade E.M., Beynon S.A., Lewis O.T. (2016). Functionally rich dung beetle assemblages are required to provide multiple ecosystem services. Agric. Ecosyst. Environ..

[66] Hardersen S., Zapponi L. (2018). Wood degradation and the role of saproxylic insects for lignoforms. Appl. Soil Ecol..

[67] Wu L., Qing L., Yuanyuan W., Longyu Z., Yanlin Z., Ziniu Y., Huanchun C., Jibin Z. (2018). Efficient bioconversion of organic wastes to value-added chemicals by soaking, black soldier fly (*Hermetia illucens* L.) and anaerobic fermentation. J. Environ. Manag..

[68] Lijie Y., Xiangfang Z., Shiyan Q. (2021). Advances in research on solid-state fermented feed and its utilization: The pioneer of private customization for intestinal microorganisms. Anim. Nutr..

[69] Xiang Z., Ju-Pei S., Chang-Long S., Sheng-Sheng J., Hong J.D., Li-Mei Z., Ji-Zheng H. (2022). Attenuation of antibiotic resistance genes in livestock manure through vermicomposting via *Protaetia brevitarsis* and its fate in a soil-vegetable system. Sci. Total Environ..

[70] Anshu S., Satyawati S. (2002). Composting of a crop residue through treatment with microorganisms and subsequent vermicomposting. Bioresour. Technol..

[71] Shweta K.R., Singh B.L., Deepshikha V. (2010). Integrating microbial composting and vermicomposting for effective utilization of by-products of sugar cane–processing industries. Bioremediat. J..

[72] Moran-Salazar R.G., Marino-Marmolejo E.N., Rodriguez-Campos J., Davila-Vazquez G., Contreras-Ramos S.M. (2016). Use of agave bagasse for production of an organic fertilizer by pretreatment with *Bjerkandera adusta* and vermicomposting with *Eisenia fetida*. Environ. Technol..

[73] Kashif-ur R., Rashid U.R., Abdul A.S., Minmin C., Longyu Z., Xiaopeng X., Asif U.R., Abdul R., Jeffery K.T., Ziniu Y. (2019). Enhanced bioconversion of dairy and chicken manure by the interaction of exogenous bacteria and black soldier fly larvae. J. Environ. Manag..

[74] Kui W., Peiwen G., Lili G., Chunqin L., Jie Z., Changlong S. (2022). Lignocellulose degradation in *Protaetia brevitarsis* larvae digestive tract: Refining on a tightly designed microbial fermentation production line. Microbiome.

[75] Baohai D., Huina X., Lili G., Weihang L., Jie Z., Wensheng X., Rongmei L., Changlong S. (2022). Microflora for improving the *Auricularia auricula* spent mushroom substrate for *Protaetia brevitarsis* production. iScience.

[76] Fuqing G. (2014). Research on the Effect of Different C/N Ratios on Fermentation of Organic Fertilizer. Master’s Thesis.

[77] Takahashi N., Mochizuki S., Masuda K., Shimada I., Osada M., Fukunaga H. (2017). Influence of temperature, water content and C/N ratio on the aerobic fermentation rate of woody biomass. Kagaku Kogaku Ronbunshu.

[78] Carotenuto C., Guarino G.D., Amelia L.I., Morrone B., Minale M. (2020). The peculiar role of C/N and initial pH in anaerobic digestion of lactating and non-lactating water buffalo manure. Waste Manag..

[79] Tao X. (2020). Technical Research on *Protaetia brevitarsis* Lewis Bioconversion of Cattle Farm Waste in Indoor and Outdoor. Master’s Thesis.

[80] Yusheng L. (2012). Insect Production Science.

[81] Xiaofang Z., Liuyang W., Chunqin L., Yongqiang L., Xiangdong M., Zhongyue W., Tao Z. (2021). Identification and field verification of an aggregation pheromone from the white-spotted flower chafer, *Protaetia brevitarsis* Lewis (Coleoptera: Scarabaeidae). Sci. Rep..

[82] Doube B.M. (2018). Ecosystem services provided by dung beetles in Australia. Basic Appl. Ecol..

[83] Gossner M.M., Lachat T., Brunet J., Isacsson G., Bouget C., Brustel H., Brandl R., Weisser W.W., Muller J. (2013). Current near-to-nature forest management effects on functional trait composition of saproxylic beetles in beech forests. Conserv. Biol..

[84] Pardillo N. (2018). Production efficiency of organic fertilizer from different composting methods. Asia Pac. J. Multidiscip. Res..

[85] Yingkai L., Jiali L., Xiyue S., Yali W., Xiaolei Y., Wen G., Yinsheng L. (2020). Effect of adding cow dung and garden waste on sewage sludge vermicomposting process. Chin. J. Environ. Eng..

[86] Lordelo M.M., Calhoun M.C., Dale N.M., Dowd M.K., Davis A.J. (2007). Relative toxicity of gossypol enantiomers in laying and broiler breeder hens. Poult. Sci..

[87] Yunfeng L., Xiuqi W., Qingyu Z., Zhang Junmin Z. (2010). Research situation on gossypol safety limit in feed and gossypol residues in livestock product. Chin. Agric. Sci. Bull..

[88] Rehemujiang H., Yimamu A., Wang Y.L. (2019). Effect of dietary cotton stalk on nitrogen and free gossypol metabolism in sheep. Asian-Australas J. Anim. Sci..

[89] Wenju Z., Zirong X., Shunhong Z., Jianyi S., Xia Y. (2007). Development of a microbial fermentation process for detoxification of gossypol in cottonseed meal. Anim. Feed Sci. Technol..

[90] Vellaichamy M., Sharmila B.M., Kuppusamy P. (2017). Isolation and identification of potential Gossypol degrading fungal strains from cotton growing soil. Int. J. Microbiol..

[91] Xiuye Q., Quanxi X., Jiamin Y., Qian Z., Zhiyan Z., Haiyan X., Wei G. (2019). Screening of free gossypol strain in high efficient degrading cottonseed meal and optimization of compound fermentation. J. Chin. Cereals Oils Assoc..

[92] Van Huis A. (2013). Potential of insects as food and feed in assuring food security. Annu. Rev. Entomol..

[93] Choi S.U., Choi I.H., Chung T.H. (2021). Investigation of breast meat traits of broilers fed different amounts of *Hermetia illucens* and *Protaetia brevitarsis seulensis* powder. Entomol. Res..

[94] Deokyeol J., Namgyong M., Yeongbu K., Soo-Rin K., Ohseok K. (2019). The effects of feed materials on the nutrient composition of *Protaetia brevitarsis* larvae. Entomol. Res..

[95] Quan W., Zhen W., Mukesh K.A., Yahui J., Ronghua L., Xiuna R., Junchao Z., Feng S., Meijing W., Zengqiang Z. (2016). Evaluation of medical stone amendment for the reduction of nitrogen loss and bioavailability of heavy metals during pig manure composting. Bioresour. Technol..

[96] Malinska K., Golanska M., Caceres R., Rorat A., Weisser P., Slezak E. (2017). Biochar amendment for integrated composting and vermicomposting of sewage sludge the effect of biochar on the activity of *Eisenia fetida* and the obtained vermicompost. Bioresour. Technol..

[97] Yan H., Rong L., Hongjun L., Beibei W., Chenmin Z., Qirong S. (2014). Novel resource utilization of refloated algal sludge to improve the quality of organic fertilizer. Environ. Technol..

[98] Yajuan C., Ji L., Yaofeng Y. (2016). Dynamic change of key indicators and denitrifying bacteria in chicken manure sawdust aerobic composting process. J. Chin. Agric. Univ..

[99] Xiuhong W., Xiangyuan S., Jitao Z., Yuxia W., Xinxin L., Jing Z., Hongye Z. (2021). Analysis of maturity, heavy metal residues and microbial flora of chicken manure aerobic compost. Shanxi Agric. Sci..

